# Grifolin directly targets ERK1/2 to epigenetically suppress cancer cell metastasis

**DOI:** 10.18632/oncotarget.5678

**Published:** 2015-10-26

**Authors:** Xiangjian Luo, Lifang Yang, Lanbo Xiao, Xiaofeng Xia, Xin Dong, Juanfang Zhong, Ying Liu, Namei Li, Ling Chen, Hongde Li, Wei Li, Wenbin Liu, Xinfang Yu, Hanyong Chen, Min Tang, Xinxian Weng, Wei Yi, Ann Bode, Zigang Dong, Jikai Liu, Ya Cao

**Affiliations:** ^1^ Cancer Research Institute, Key laboratory of Chinese Ministry of Education, Xiangya School of Medicine, Central South University, Changsha, Hunan 410078, PR China; ^2^ Molecular Imaging Center, Xiangya Hospital, Central South University, Changsha, Hunan 410078, PR China; ^3^ Department of Systems Medicine and Bioengineering, Houston Methodist Research Institute, Houston, TX 77030, USA; ^4^ The Hormel Institute, University of Minnesota, Austin, MN 55912, USA; ^5^ State Key Laboratory of Phytochemistry and Plant Resource in West China, Kunming Institute of Botany, Chinese Academy of Sciences, Kunming, Yunnan 650204, PR China

**Keywords:** grifolin, ERK1/2, DNMT1, metastasis

## Abstract

Grifolin, a secondary metabolite isolated from the fresh fruiting bodies of the mushroom *Albatrellus confluens*, has been reported by us and others to display potent antitumor effects. However, the molecular target of grifolin has not been identified and the underlying mechanism of action is not fully understood. Here, we report that the ERK1/2 protein kinases are direct molecular targets of grifolin. Molecular modeling, affinity chromatography and fluorescence quenching analyses showed that grifolin directly binds to ERK1/2. And *in vitro* and *ex vivo* kinase assay data further demonstrated that grifolin inhibited the kinase activities of ERK1/2. We found that grifolin suppressed adhesion, migration and invasion of high-metastatic cancer cells. The inhibitory effect of grifolin against tumor metastasis was further confirmed in a metastatic mouse model. We found that grifolin decreased phosphorylation of Elk1 at Ser383, and the protein as well as the mRNA level of DNMT1 was also down-regulated. By luciferase reporter and ChIP assay analyses, we confirmed that grifolin inhibited the transcription activity of Elk1 as well as its binding to the *dnmt1* promoter region. Moreover, we report that significant increases in the mRNA levels of *Timp2* and *pten* were induced by grifolin. Thus, our data suggest that grifolin exerts its anti-tumor activity by epigenetic reactivation of metastasis inhibitory-related genes through ERK1/2-Elk1-DNMT1 signaling. Grifolin may represent a promising therapeutic lead compound for intervention of cancer metastasis, and it may also be useful as an ERK1/2 kinase inhibitor as well as an epigenetic agent to further our understanding of DNMT1 function.

## INTRODUCTION

In recent years, natural agents such as the pool of secondary metabolites produced by a variety of mushrooms, has attracted a great deal of chemopreventive and therapeutic interest [1]. These compounds originate as derivatives from many intermediates in primary metabolism. Grifolin, a farnesyl phenolic compound, is a secondary metabolite originating from the fresh fruiting bodies of the mushroom *Albatrellus confluens*. It is also reportedly isolated from the edible mushroom *Boletus pseudocalopus* [2]. Grifolin has shown various microbiological and pharmacological effects [3–7]. The anticancer activities of grifolin were first reported by our group [8] and those previous studies showed that grifolin inhibits the growth of cancer cell lines by inducing of apoptosis and G1-phase cell-cycle arrest [9–11]. Another study also demonstrated that grifolin induces apoptosis in human osteosarcoma cells [12].

The ERK1/2 signaling consists of a three-tier hierarchical cascade of protein kinases including RAF, MEK1/2 (mitogen-activated protein kinase kinase1/2) and ERK1/2(extracellular signal-regulated kinase1/2). The ERKs cascade is frequently de-regulated in approximately one-third of all human cancers [13–15]. And it is closely associated with tumor cell inflammation, migration, invasion and metastasis, suggesting that suppression of ERKs signaling may represent a potential approach for preventing cancer metastasis [15, 16].

Tumor cells require genetic and/or epigenetic changes in order to be transformed into metastatic cells. DNA methylation performed by a family of DNA methyltransferase (DNMT) enzymes, plays a critical role in epigenetic gene regulation in many forms of cancer [17–19]. And aberrant hypermethylation of a significant number of metastasis suppressor-related genes, as tissue inhibitors of proteinases (i.e. TIMPs), cadherins, etc., have been widely investigated. Epigenetic interference of hypermethylated tumor suppressor genes has been gaining great attention as promising anticancer targets [20].

Here, we reports that grifolin inhibits the kinase activity of ERK1/2 by physically binding to the respective-ATP pocket and impeding the downstream signaling. The inhibition of the ERK1/2-Elk1-DNMT1 pathway by grifolin led to the restoration of the function of metastasis suppressor-related genes, *Timp2* and *pten*, which interfered the motility, invasion and metastasis of cancer cells.

## RESULTS

### Grifolin physically binds to ERK1/2 to inhibit ERK1/2 kinase activities

**Figure 1:**
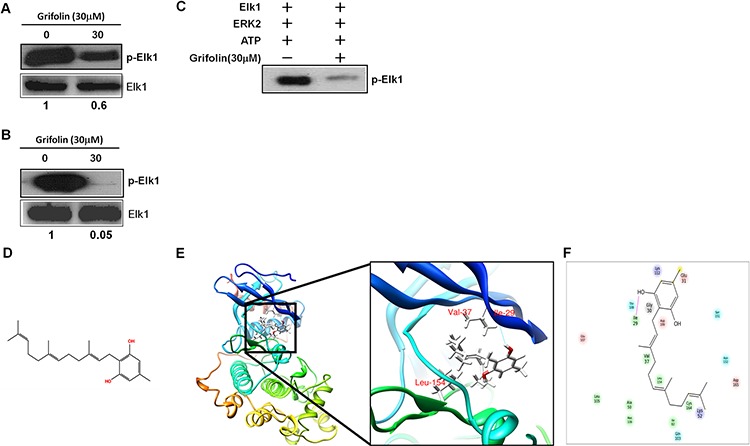
Molecular modeling of grifolin binding to ERK2 Grifolin inhibits the *ex vivo* kinase activity of ERK1/2 in **A.** MCF7 and **B.** HeLa cells. **C.** Grifolin inhibits the kinase activity of ERK2 *in vitro*. **D.** Chemical structure of grifolin. **E.** Grifolin binds to the ATP pocket of ERK2. The box indicates an enlarged view. Hydrogen bonds are formed between grifolin and Ile-29 at the backbone of ERK2. **F.** Ligand interaction diagram of the ERK2 and grifolin complex. Residues are represented as colored spheres and are labeled with the residue's name and number. The colors indicate the residue type (green: hydrophobic; blue: polar). The solid pink line shows the hydrogen bond between the ligand and the receptor. Hydrophobic interactions are formed at Val-37 and Leu-154.

In previous studies, we found that grifolin suppressed ERK1/2 kinase activity in a dose-dependent manner in the CNE1 nasopharyngeal carcinoma cell line [9]. This effect was further confirmed in MCF7 human breast cancer cell line (Figure 1A) and the HeLa human cervical cancer cell line(Figure 1B). To determine whether grifolin could inhibit ERK2 kinase activity in a cell-free system, active recombinant ERK2 was added with ATP and an Elk-1 fusion protein in kinase buffer, Elk-1 phosphorylation was then detected by Western blot using the phospho-Elk-1(Ser383) antibody. Grifolin significantly inhibited ERK2 kinase activity *in vitro* (Figure 1C). Collectively, these observations indicate that grifolin inhibits the kinase activity of EKR1/2 in cells and *in vitro*, which prompted us to determine grifolin's mechanism of action against ERK1/2.

**Figure 2:**
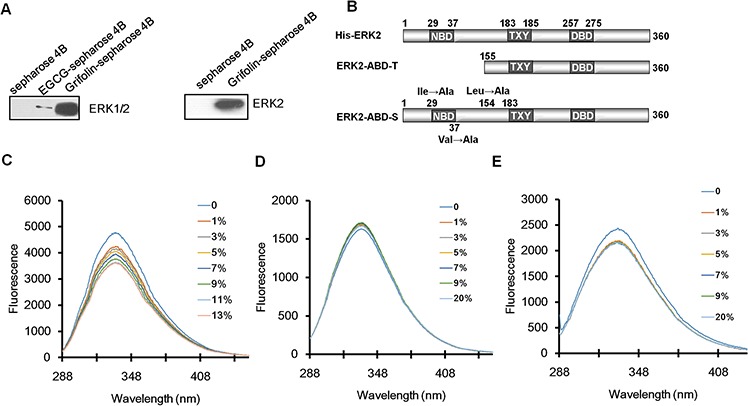
Grifolin physically binds to ERK1/2 **A.** Immunoblot analysis of whole cell lysate following purification by a Sepharose 4B, grifolin-Sepharose 4B or EGCG-Sepharose affinity column. The ERK2/grifolin complex was confirmed by Western blot using an antibody against ERK1/2 (*left panel*). A grifolin-Sepharose 4B or Sepharose 4B affinity column was each incubated with recombinant active ERK2. After washing, the active ERK2/grifolin complex was confirmed by Western blot (*right panel*). **B.** Schematic representations of ERK2 protein domains and truncation and site mutant proteins. NBD, ATP binding domain. DBD, DNA binding domain. Effect of grifolin on fluorescence quenching of **C.** ERK2 and **D.** truncation ERK2-ABD-T and **E.** site mutant ERK2-ABD-S.

We next used molecular modeling with the crystal structure of ERK2 to determine whether grifolin could bind to ERK2. To examine how grifolin combines with ERK2, we docked it *in silico* to the ATP binding pocket of ERK2 using several protocols in Schrödinger Suite 2011. We found that grifolin formed an important hydrogen bonds with Ile-29 at the backbone of ERK2, and also formed hydrophobic interactions with ERK2 at Val-37 and Leu-154 (Figures 1E–1F). The molecular modeling results of ERK1/2 infer a physical binding of grifolin to ERK1/2. We thus tested this possibility using a number of methods. First, we used affinity chromatography analysis to determine whether grifolin binds to the ERK1/2 proteins. Lysates prepared from CNE1 cells were incubated with affinity columns of grifolin-Sepharose 4B, EGCG-Sepharose 4B (as a positive control, which had been reported in our previous study [21]) or Sepharose 4B beads (as a negative control). And the pulled-down proteins were analyzed by Western blot. We found that CNE1 cell lysates containing ERK1/2 combined with grifolin-Sepharose 4B as well as EGCG-Sepharose 4B, but not Sepharose 4B beads (Figure 2A, *left*). And nor Akt/grifolin or p38/grifolin complexes was detected by Western blot (Supplementary Figure S1). All the data demonstrated that grifolin can bind with ERK1/2 in cells, and the binding has certain specificity. Furthermore, to assess the significance of grifolin binding with ERK1/2, we used a cell-free system and Sepharose 4B beads as a negative control. Results indicated that commercially available active ERK2 did not combine with the Sepharose 4B affinity column, but did bind with grifolin-Sepharose 4B (Figure 2A, *right*).

Secondly, we investigated which domain of ERK2 was involved in grifolin binding by generating a truncation mutant, ERK2-ABD-T, in which the ATP binding pocket was truncated and the skeleton of ERK2 remained. We also constructed site mutants of ERK2 (ERK2-ABD-S), in which Ile29, Val37 and Leu154 were each mutated to Ala (Figure 2B). We then studied the physical interaction between grifolin and ERK1/2 using fluorescence quenching analysis. The His-fusion wild-type EKR1/2 proteins displayed maximal fluorescence at 334 nm (Figure 2C). When the His-ERK2 protein was incubated with increasing concentrations of grifolin, the fluorescence intensity gradually decreased. The fluorescence intensity of ERK2-ABD-T (Figure 2D) and ERK2-ABD-S (Figure 2E) did not change significantly with increasing amounts of grifolin. Taken together, the data indicate that grifolin physically binds to the ATP-pocket of the ERK2 protein to inhibit ERK2 kinase activity.

### Grifolin suppresses motility and invasion of metastatic carcinoma cells

**Figure 3:**
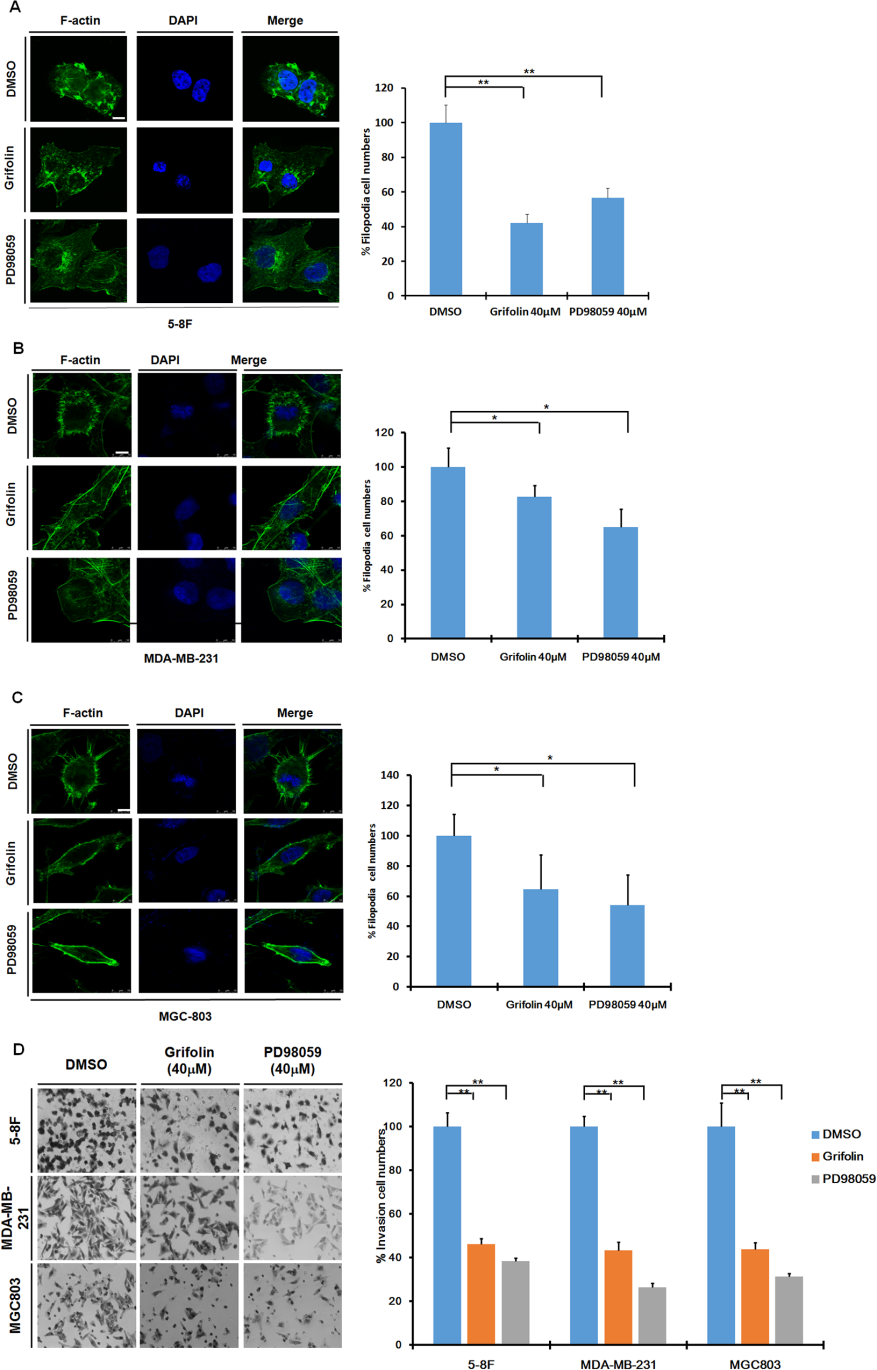
Grifolin Inhibits motility and invasion of high-metastatic cancer cells Grifolin inhibits filopodia formation in **A.** 5–8F, **B.** MDA-MB-231 and **C.** MGC803 cells. Cells were treated with 0.1% DMSO (control), 40 μM grifolin or 40 μM PD98059 for 24 h. Cells were then stained with fluorescein isothiocyanate/phalloidin and visualized using laser-scanning confocal microscopy. Images are representative of at least 3 independent experiments. Data are shown as mean values ±S.D. of filopodia counted from 5 fields. The asterisks (*,**) indicate a significant difference (*p* < 0.05, *P* < 0.01, respectively) compared to untreated control. Scale bar, 50 μm. **D.** Grifolin inhibits tumor cell invasion. Highly metastatic 5–8F, MDA-MB -231 or MGC803 cells were treated with grifolin at 40 μM or PD98059 at 40 μM for 48 h and allowed to invade through Matrigel-coated chambers toward an attractant of 10% serum media. Counts were obtained (in triplicate fields of view) from the control DMSO group (average set at 100% invasion) and were used to calculate percent invasion for all other treatments. Data are shown as mean values ± S.D. of independent, triplicate experiments. The asterisks (**) indicate a significant difference (*P* < 0.01) compared to the untreated control.

Constitutively active ERK1/2 signaling has been shown to lead to increased tumor migration and invasion [15, 16, 22, 23], thus we further investigated the potential anti-metastatic activity of grifolin. Filopodia are associated with cell motility and adhesion [24]. To determine whether grifolin could inhibit filopodia formation, metastatic nasopharyngeal 5–8F cells were treated with 40 μM grifolin for 24 h, and filopodia formation was then observed using immunofluorescence to detect F-actin (fluorescein isothiocyanate-phalloidin). We found that microspikes aggregated on the surface of the cell and filopodia were enriched in the control; whereas in the grifolin-treated group, filopodia or microspike structures markedly decreased (Figure 3A). This was further investigated in the highly metastatic human breast cancer cell line MDA-MB-231 (Figure 3B) and in the gastric cancer cell line MGC803 (Figure 3C). Compared with the control, grifolin treatment significantly reduced filopodia in 5–8F, MDA-MB-231 and MGC803 cells. Similar results were observed after cells were treated with 40 μM PD98059 for 24 h. In the meanwhile, grifolin remarkably decreased phosphor-ERK level in high-metastatic tumor cells (Supplementary Figure S2). Furthermore, we introduced mutant constitutively active MEK1 or MEK2 into 5–8F cells, and found both rescued the filopodia formation suppressed by grifolin treatment(Supplementary Figure S3A). All these data further correlate the morphology change and inhibition of ERK1/2 signaling. It indicates that grifolin suppresses the motility of metastatic carcinoma cells through inhibition of the ERK1/2 pathway.

Aberrant invasion is one of the main processes by which cancer cells evade tissue boundaries to achieve colonization at metastatic sites. To investigate the anti-invasion effect of grifolin, the infiltration of 5–8F cells through Matrigel in a Boyden chamber assay was measured. Results demonstrated that after 48 h treatment with 40 μM grifolin, the number of cells that invaded into the lower chamber was significantly reduced by 46.16%. Similar inhibitory effects were also observed in MDA-MB-231 and MGC-803 cells (43.4% and 43.74% inhibition, respectively) compared to the DMSO control (Figure 3D). The reintroduction of active MEK1 or MEK2 into 5–8F cells reversed the cell invasion capacity inhibited by grifolin (Supplementary Figure S3B), which indicates that grifolin exerts the anti-invasive activity by impeding ERK1/2 pathway.

### Grifolin exerts anti-metastatic effects *in vivo*

**Figure 4:**
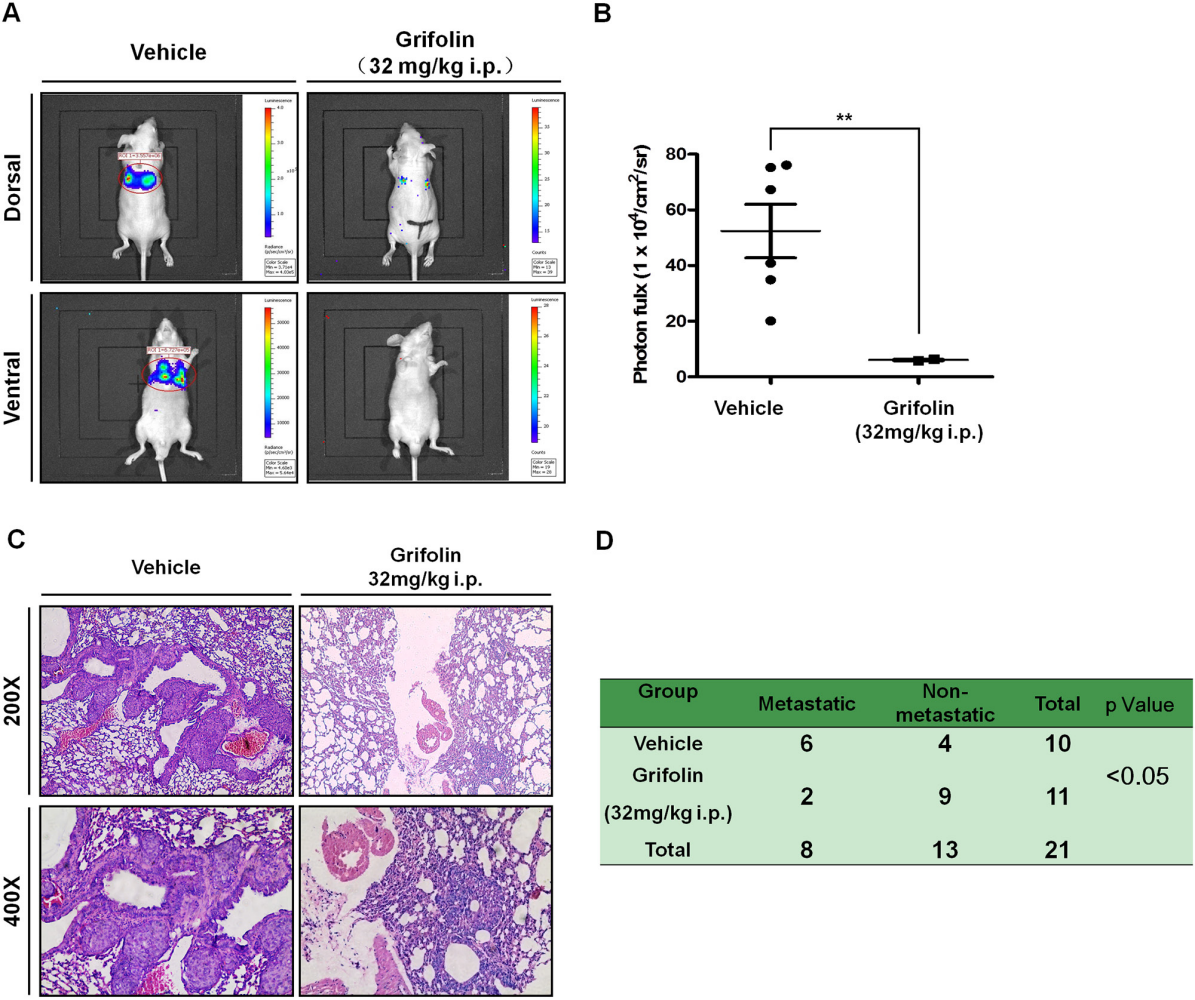
Grifolin inhibits tumor metastasis *in vivo* 5–8F-Z cells stably expressing luciferase were implanted into nude mice by tail vein inoculation. These same mice were treated with vehicle (corn oil) or 32mg/kg/day of grifolin for 25 days and then animals were imaged using the IVIS Lumina II Imaging System. **A.** Representative images of control or grifolin-treated mice inoculated with 5–8F cells and the corresponding lung metastasis development. **B.** Scatter plot of lung photon flux for 5–8F-Z-injected mice. The relative lung metastasis in the control and grifolin-treated group is noted for each animal. The asterisks (**) indicate a significant difference (*p* < 0.01). **C.** H&E staining of lung metastasis in mice at 200 × or 400× magnification. **D.** The metastatic rate of control and grifolin-treated groups was analyzed by Chi-square test (*p* < 0.05).

To evaluate the effects of grifolin on tumor metastasis *in vivo*, we first established stable expression of luciferase in the highly metastatic nasopharyngeal carcinoma cell line 5–8F (Supplementary Figure S4). Then 5–8F-Z cells were injected into the tail veins of BABL/c nude mice to establish a visualazable metastatic mouse model. These same mice were treated with grifolin (32mg/kg/day) for 25 days, and metastsis was assessed. Bioluminescence imaging (Figures 4A–4B) and hematoxylin-&eosin (H&E) staining (Figure 4C) of lung tissue confirmed that the incidence of metastasis was 60% (6/10) in the control group. In contrast, in the grifolin-treated group, metastasis significantly decreased to 18.2% (2/11, *p* < 0.05, Chi-square; Figure 4D). Importantly, grifolin did not cause any adverse effects compared with the control. The results indicated that the metastatic activity of 5–8F-Z cells to the lung was significantly abated by grifolin treatment.

### Grifolin inhibits ERK1/2-Elk1-DNMT1 signaling in metastatic carcinoma cells

**Figure 5:**
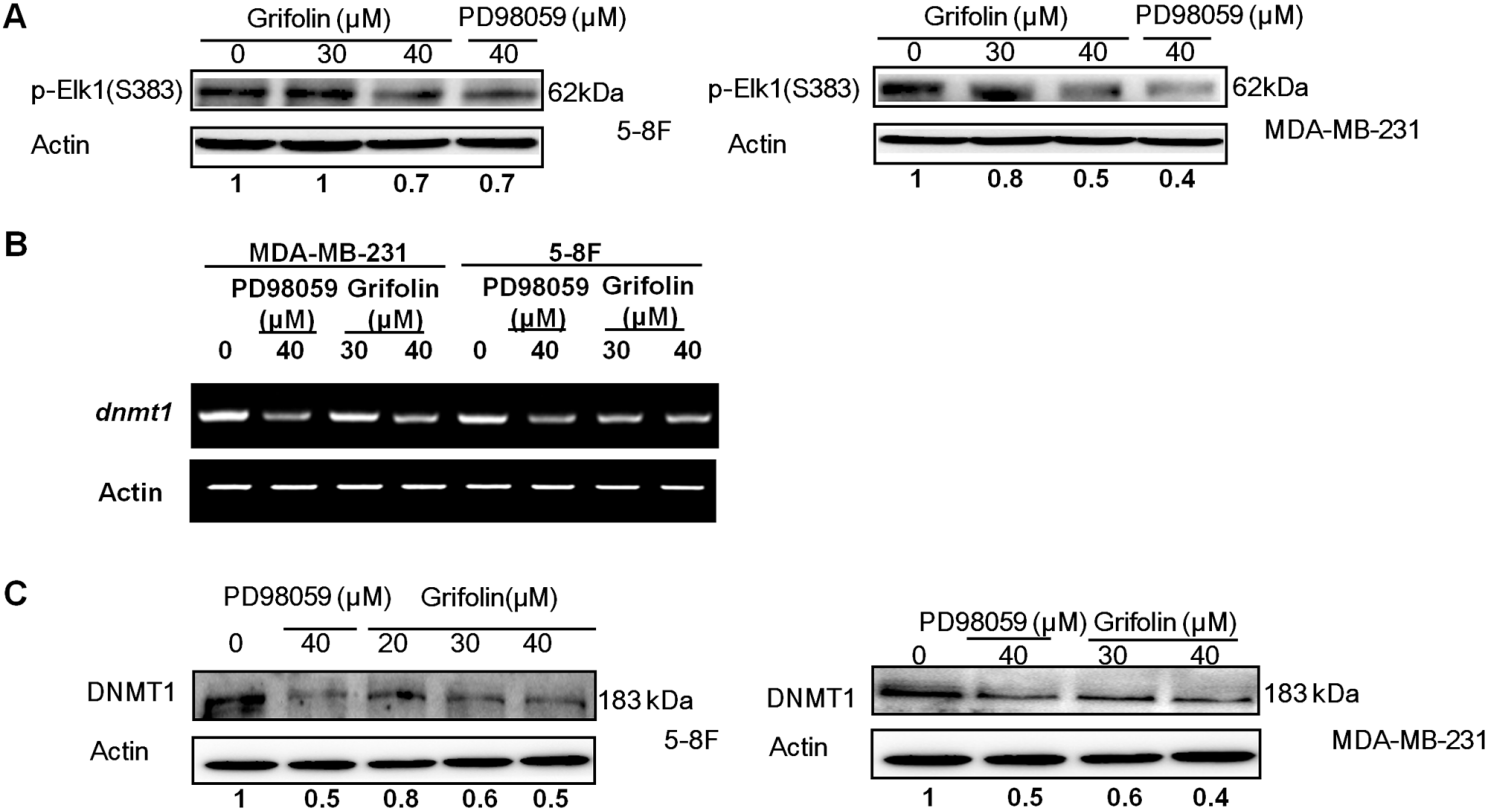
Grifolin decreases ERK1/2 downstream signaling **A.** 5–8F or MDA-MB-231 cells were treated with either DMSO (control) or various concentrations of grifolin for 12 h. Cell lysates were prepared and examined by Western blot with an antibody to detect phosphorylated Elk1 (Ser383). **B.** Grifolin attenuates dnmt1 mRNA levels. 5–8F or MDA-MB-231 cells were treated with either DMSO or various concentrations of grifolin for 24 h. Total RNA was isolated from cells and subjected to RT-PCR. The dnmt1 PCR primers were: sense 5′-GGA AAGGAGGAGACT CT AC-3′; antisense: 5′-TCTCACTTGCCACCC ACACA-3′ **C.** Grifolin inhibits DNMT1 protein expression. Cells were cultured and treated with DMSO or grifolin for 24 h and DNMT1 was detected by Western blot using a specific antibody. Actin served as a loading control.

To further explore the action mechanism of grifolin on ERK1/2 pathway, we examined Elk1, which is a downstream element of ERK1/2 signaling. Phosphorylation at S383 is prerequisite for activation of Elk1 transcription activity. We demonstrated that grifolin decreased the phosphorylation of Elk1(S383) in 5–8F and MDA-MB-231 cells compared to the untreated control (Figure 5A). Treatment with grifolin attenuated the mRNA level of *dnmt1* in a dose-dependent manner (Figure 5B). Inhibition of DNMT1 expression was further confirmed at the protein level by Western blot (Figure 5C).

**Figure 6:**
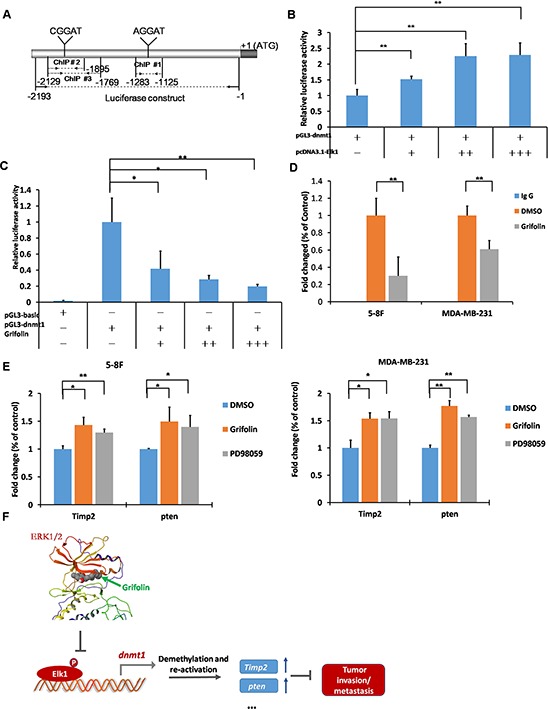
Grifolin suppresses ELK1-DNMT1 signaling to demethylate and restore epigenetically silenced metastatic suppressor genes **A.** Schematic of the *dnmt1* promoter highlighting the regions used in the dual-luciferase and ChIP assay as well as the consensus sequences (A/CGGAA/T) present. **B.** The enhancement of *dnmt1* promoter activity as a result of increased Elk1 expression. HEK293T cells were transfected with pcDNA3.1-Elk1, pGL3-dnmt1, and pRL-TK as an internal control for transfection efficiency. Cells were harvested at 48 h after transfection and subjected to the luciferase assay. **C.** The *dnmt1* promoter activity was suppressed by grifolin in a dose-dependent manner. HEK293T cells were transfected with pGL3-basic or pGL3-dnmt1 and pRL-TK as an internal control. At 24 h after transfection, cells were treated with increasing concentrations of grifolin (0–40 μM) as indicated for another 24 h, then cells were harvested and subjected to the luciferase assay. **D.** Grifolin inhibits Elk1 binding to the *dnmt1* protmoter region. ChIPs were performed in 5–8F and MDA-MB-231 cells with different antibodies: anti-Elk1 or anti-IgG (negative control). The *dnmt1* of metastasis suppressor-related genes promoter region was amplified by real-time PCR. **E.** Restored mRNA expression of metastasis suppressor-related genes *Timp2* and *pten* induced by grifolin. 5–8F or MDA-MB-231 cells were treated with DMSO, 40 μM grifolin or 40 μM PD98059 as designated for 24 h. Total RNA was isolated from cells and subjected to real-time PCR. PCR primers were as follows: *Timp2*, sense 5′-ATAAGCA GGCCTCCAACGC-3′; antisense 5′-GAGCTGGACCAGTCGAAACC-3′; *pten*, sense 5′-TGAGTTCCCTCAGCC GTTACCT-3′; antisense 5′-GAGGTTTCCTCT GGTCCTGGTA-3′. Data are shown as mean values ± S.D. and the asterisks (*,**) indicate a significant difference (*p* < 0.05, *p* < 0.01, respectively) compared to the DMSO control. **F.** Schematic illustration of the suppression of ERK1/2-ELK1-DNMT1 signaling by grifolin to demethylate and restore epigenetically silenced metastatic suppressor genes in cancer cells.

Elk1 is a transcription factor belonging to the Ets family and is capable of directly binding to consensus sites (5′-A/CGGAA/T -3′) to exert transcriptional effects. Several potential binding sites for Elk1 are present in the *dnmt1* proximal promoter (Figure 6A). To determine the role of Elk1 in the transcriptional regulation of *dnmt1*, we used a reporter construct containing a 2193bp fragment of the *dnmt1* promoter and increasing concentrations of Elk1 expression in dual-luciferase reporter assays in HEK293T cells. We found that augmentation of Elk1 increased *dnmt1* promoter activity in a dose-dependent manner (Figure 6B), which showed that Elk1 protein binding to this site promoted the induction of *dnmt1* transcription. Furthermore, HEK293T cells were transfected with the *dnmt1* promoter-reporter construct. After overnight incubation, cells were treated with grifolin for another 24 h followed by reporter expression analysis. In accordance with the inhibition of the *dnmt1* mRNA/protein expression, grifolin dose-dependently reduced luciferase activity driven by the *dnmt1* promoter (Figure 6C).

We next used chromatin immunoprecipitation (ChIP) to examine the influence of grifolin on the ability of Elk1to bind to the *dnmt1* promoter. In this assay, each of three potential binding sites in the *dnmt1* promoter was assessed individually for its ability to be enriched by Elk1 immunoprecipitation. The most significant site for Elk1 recruitment was chosen to apply the ChIP assay sequentially (Supplementary Figure S5). Next, chromatin was immunoprecipitated using either control rabbit IgG or an Elk1-specific antibody, and the *dnmt1* promoter region surrounding the Elk1 binding site was amplified by real-time PCR. Consistent with the luciferase reporter data, results revealed that grifolin effectively suppressed the recruitment of Elk1 to the *dnmt1* promoter. It indicates that grifolin decreases the transcription of *dnmt1* through inhibiting Elk-1 transcription activity as well as its binding to the *dnmt1* promoter region (Figure 6D).

To evaluate the function of grifolin to restrain metastasis in tumors through the inhibition of DNMT1 signaling, we focused on a subset of genes whose expression level was hypermethylated and strongly correlated with the metastatic potential of tumors. *Phosphatase and tensin homolog* (*pten*), a tumor suppressor gene, may suppress tumor cell growth by antagonizing protein tyrosine kinases and block the first step of tumor cell invasion and metastasis through its interaction with focal adhesion. CpG island hypermethylation has been identified as an alternative mechanism of PTEN inactivation in a number of human cancers [25–28]. TIMPs are a multigene family that binds noncovalently to active MMPs in a 1:1 molar ratio and inhibits their activity. Moreover, MMP/TIMP balance is a major factor in the regulation of the proteolytic activity of MMPs [29]. TIMP2 is also an endogenous inhibitor of angiogenesis that is mediated by the activation of protein tyrosine phosphatase activity [30]. The mRNA levels of *Timp2* and *pten* were markedly increased in 5–8F cells following treatment with grifolin (40 μM) and a similar effect was observed in cells treated with PD98059 (40 μM) (Figure 6E). Thus, grifolin may exert anti-metastatic effect through suppression of ERK1/2-DNMT1 signaling to restore the function of metastatic suppressor genes.

## DISCUSSSION

Extensive data are now accumulating showing that natural dietary constituents can strongly influence the potential for disease outcome [31]. Medicinal mushrooms have an established history of use in traditional oriental health care and therapy [1]. In this study, our results provide strong evidence that grifolin, a natural compound from the mushroom *Albatrellus confluens*, physically binds to the ERK1/2 protein and inhibit their kinase activity, thereby down-regulating the transcription of *dnmt1* gene by suppressing the downstream transcription factor, Elk1. The inhibition of DNMT1 by grifolin leads to reactivation of tumor-suppressor gene expression that could have an important impact on tumor invasion and metastasis.

The adopted strategy in anticancer therapeutics as well as cancer preventive agents development is based on the discovery of precise molecular targets. It successes in clinical trials and drugs such as sunitinib, sorafenib, tamoxifen [32, 33] and finasteride [33, 34]. They have been approved by the U.S. Food and Drug Administration (FDA) as cancer therapeutic or preventive agents. Indeed, the ERK1/2 pathway has attracted much interest in the search for new cancer therapeutics and several inhibitors are currently undergoing clinical evaluation. These include RAF inhibitors such as vemurafenib/PLX4032, which selectively inhibits BRAFV600E [35], and MEK1/2 inhibitors such as selumetinib (AZD6244/ARRY-142886) [36]. Yang *et al*. reported that ERK1/2 is a direct target of caffeic acid for its attenuation of solar UV-induced carcinogenesis [37].

In our previous study, we undertook a gene expression profiling analysis to identify novel targets of grifolin and found that the MAPK pathway was significantly inhibited in response to grifolin treatment [9]. *In vivo* and *in vitro* kinase assay further revealed that grifolin effectively abolished ERK1/2 kinase activity. Considering this, we docked the compound to the ATP binding site of ERK2. The hydroxyl group at the 2′ position of grifolin could form a hydrogen bond with the backbone atom of Ile29. In addition, grifolin would be sandwiched by the side chains of the hydrophobic residues in the ATP binding site including Val37 and Leu154. These hydrogen bonds and hydrophobic interactions maybe mainly contribute to the potent inhibitory activity of grifolin against ERK2. Fluorescence quenching experiments showed that grifolin quenches the fluorescence of wild-type ERK2, but not the ERK2-ABD-S mutant, or ERK2-ABD-T truncate, which indicates that grifolin physically binds to the ATP pocket of ERK2. Additional evidence was provided by affinity chromatography analysis. Therefore, for the first time, we identified ERK2 is the direct target of grifolin, to which grifolin can physically bind to and functionally inhibit its activity.

Metastasis is accompanied by various physiological alterations, such as filopodia formation, which allows cancer cells to invade the blood or lymphatic system and to spread to other tissue or organs [24]. The present study demonstrates that grifolin exert an inhibitory effect on the filopodia formation in 5–8F, MDA-MB-231 and MGC803 cells. The data from a Matrigel assay and animal experiment confirmed the inhibitory effects of grifolin. These findings provide strong evidence for the anti-invasion/metastasis effect of grifolin *in vitro* and *in vivo*.

Aberrant epigenetic alterations generally results in the repression of tumor suppression gene expression, and may enable tumor cells to metastasize or have a selective advantage at the secondary tissue site [38]. Many natural compounds, such as EGCG, resveratrol, anacardic acid, garcinol, plumbagin, curcumin and genistein can directly influence epigenetic mechanisms in humans [39–42]. Natural compounds containing phenolic compounds have been widely demonstrated to possess the capability to prevent cancer invasion/metastasis. Catechin derivatives, genistein/genistin, silibinin, quercetin, and anthocyanin have been widely investigated for their anti-metastatic activities [43, 44]. We present clear evidence that grifolin, a farnesyl phenolic compound, blocks ERK1/2-Elk1-DNMT1 signaling and reactivation the transcription of the *Timp2* and *pten* genes. Beyond this, we have screened a panel of tumor-suppressor genes in 5–8F cells and also found that the *pcdh10* and *Timp1* genes were significantly increased by grifolin (Supplementary Figure S6). Although *pcdh10* mRNA was induced by grifolin treatment, it was still very low and marginally detectable. And by bioinformatics prediction, we found that the promoter region of the *Timp1* gene lacked CpG islands, which suggested that the augmentation of *Timp1* transcription in the presence of grifolin may be DNMT1-independent. In previous study, we have shown that grifolin can inhibit tumor cell survival [8, 12], which might contribute to the suppression of metastases formation. Albeit this, here we have provided an important mechanism that grifolin blocks cancer cell invasion and metastasis by epigenetic regulation of DNMT1 function.

In summary, our study demonstrates that grifolin is an inhibitor of ERK1/2 as well as an antagonist of DNMT1. The availability of this natural product will represent a promising candidate lead compound in the intervention of cancer invasion/metastasis by impeding ERK1/2-Elk1-DNMT1 signaling.

## MATERIALS AND METHODS

### Cell culture

The human nasopharyngeal carcinoma CNE1 and 5–8F, human gastric MGC803, human breast MCF7 and human cervical cancer HeLa cell lines were grown in RPMI 1640 media and the human breast MDA-MB-231(ATCC HTB-26) cancer cell line was grown in DMEM. All were supplemented with 10% v/v heat-inactivated foetal bovine serum(FBS), 1% w/v glutamine and 1% w/v antibiotics and cultured at 37°C in a humidified incubator containing 5% CO_2_. The 5–8F, MGC803 and MDA-MB-231 cell lines are highly metastatic [45].

### Reagents and chemicals

The antibodies against ERK1, p38, Elk1 and phosphor-ERK were obtained from Santa Cruz Biotechnology. The antibody against DNMT1 was from Epitomics. The antibodies against phosphor-Elk1 (Ser383), Akt, and p44/42 MAP kinase assay kit were purchased from Cell Signaling Technology. CNBr-Sepharose 4B were purchased from Amersham Pharmacia Biotech. PD98059 was obtained from Calbiochem.

The coding region of human Elk-1 was cloned by PCR and inserted into pcDNA3.1 vector to construct the eukaryotic expression Elk-1 plasmid pcDNA3.1-Elk1. The 2193 bp *dnmt1* promoter fragment was inserted into pGL3-Basic vector (Promega) and the plasmid was designated as pGL3-dnmt1. The full-length coding region of MEK1 with mutations(S218,S222) or MEK2 with mutations(S222,S226) was inserted into GV141 vector by XhoI and KpnI to construct the eukaryotic expression constitutively active MEK1 and MEK2 plasmids, respectively.

Grifolin (2-trans, trans-farnesyl-5-methylresorcinol) was provided by Kunming Institute of Botany, the Chinese Academy of Sciences (purity > 99%, HPLC analysis). Dimethyl sulphoxide (DMSO, Sigma) was used to dissolve grifolin. The final concentration of DMSO in the culture media was kept less than 0.1% v/v which had no significant effect on the cell growth.

### Molecular modeling

First the three-dimensional (3D) structure of ERK2 was obtained from the Protein Data Bank (PDB ID: 2OJJ). It is an X-ray structure with a 2.40 Å resolution of the ERK2 in complex with (S)-N-(1-(3-chloro-4-fluorophenyl) -2-hydroxyethyl) -4-(4-(3-chlorophenyl) -1H-pyrazol-3-yl) -1H-pyrrole-2-carboxamide [46]. The protein was prepared for docking by using the Protein Preparation Wizard in Schrodinger Suite 2011 [47]. All crystallographic waters were deleted and a 30-Å3 grid was generated based on the ATP binding site of the protein receptor. Grifolin was prepared using LigPrep of Schrodinger Suite 2011 under the OPLS_2005 force field and a specifying pH value of 7.0. Several standard procedures of Schrödinger's GLIDE docking protocols were performed. Procedures included docking with standard precision (SP) or extra precision (XP) in GLIDE, and the more CPU-intensive Induced-Fit Docking (IFD) method with the default parameters, which were conducted with SP and XP docking. All these docking procedures allowed ligand docking flexibility and a total of 20 top ranked structures were analyzed in IFD.

### *In vitro* kinase assay

Elk1(1 μg) was used as the substrate for an *in vitro* kinase assay with 100 ng of ERK2. Reactions were carried out in 1 × kinase buffer [25 mM Tris-HCl pH 7.5, 5 mM β-glycerophosphate, 2 mM dithiothreitol (DTT), 0.1 mM Na_3_VO_4_, 10 mM MgCl_2_, and 5 mM MnCl_2_] containing 100 μM ATP at 30°C for 30 minutes. Reactions were stopped and phosphorylated Elk1 was detected by Western blot.

### *In vitro* and *ex vivo* pull-down assays

Recombinant ERK2 or a cellular supernatant fraction (200 μg protein) was incubated with the grifolin-Sepharose 4B or Sepharose 4B only as control beads(50 μL, 50% slurry) in reaction buffer (50 mM Tris-HCl pH 7.5, 5 mM EDTA, 150 mM NaCl, 1 mM DTT, 0.01% NP40, 2 μg/mL bovine serum albumin, 0.02 mM PMSF, 1 × protease inhibitor mixture). After incubation with gentle rocking overnight at 4°C, the beads were washed 5 times with buffer(50 mM Tris-HCl pH 7.5, 5 mM EDTA, 150 mM NaCl, 1 mMDTT, 0.01% NP40, 0.02 mM PMSF), and proteins bound to the beads were analyzed by immunoblotting.

### Fluorescence quenching assay

Fluorescence spectra were measured as previously described [48, 49] using a fluorescence spectrophotometer (model F-4500, HITACHI). A His ERK2 fusion protein, truncation mutant ERK2-ABD-T and site mutant ERK2-ABD-S were expressed in the BL21 *E. coli* strain, followed by purification using Ni-NTA (nickel-nitrilotriacetic acid, Qiagen). The purified proteins were dialyzed against PBS. The His-fusion proteins were incubated with different concentrations of grifolin. Protein quenching was monitored at 25°C by using 5 nm of excitation and 5 nm of emission slit-width. The excitation wavelength was 280 nm, and the emission spectra were measured between 285 and 450 nm.

### Immunofluorescence analysis

Cells were fixed with 2.0% formaldehyde in PBS for 30 min, washed with PBS three times, and treated with PBS containing 0.2% Triton X-100 for 10 min, followed by incubation with 0.5% bovine serum albumin in PBS. Then cells were stained with 5 μg/ml fluorescein isothiocyanate-phalloidin (Sigma) for 20 min and examined using TCS SP5 confocal microscope (Leica, Germany). Random fields were counted for cells with filopodia.

### Cell invasion assay

Cells were treated with the indicated concentration of grifolin for 24 h. The inside of 8.0 μm pore-size cell culture inserts (BD Biosciences) were coated with 0.75 mg/ml Matrigel (BD Biosciences) for 1 h. 5.0 × 104 cells in serum-free media were placed inside each chamber. Cells were allowed to invade for 48 h towards an attractant of 10% fetal bovine serum. Chamber filters were fixed in buffered formalin and stained with crystal violet. Random fields were counted for invading cells under a light microscope.

### Evaluation of anti-metastatic acitivity of grifolin in nude mice

Animal procedures were in accordance with the standards established by the Guidelines for the Care and Use of Laboratory Animals of Central South University. The 5–8F cell lines was engineered to stably express the pLV.EX3d.P vector encoding luciferase. Cell suspensions (10^5^ cells in 0.1ml PBS) were injected into the lateral tail vein of nude mice. The experimental group of nude mice was administered grifolin once daily at a concentration of 32mg/kg body weight for 25 days starting on day 1 after the cell injection. The control group was injected with the corresponding corn oil solvent. Following intraperitoneal injection with D-luciferin (150mg/kg), mice were exposed to bioluminescence using the IVIS Lumina II (Xenogen). Incidence of metastasis was quantified on the basis of the luminescent signal in lung on day 25. The present study protocols were approved by the ethics committee of the Xiangya Medical School of Central South University.

### Statistical analysis

All statistical calculations were performed with the statistical software program SPSS ver.16.0. Differences between various groups were evaluated by a Student's t test or a Chi-squared test and a *p* value < 0.05 was considered statistically significant.

## SUPPLEMENTARY FIGURES


