# Supplementary material for: Grifolin directly targets ERK1/2 to epigenetically suppress cancer cell metastasis
Source: Oncotarget. 2015 Oct 26;6(40):42704–16. doi: 10.18632/oncotarget.5678 (PMC4767464; doi:10.18632/oncotarget.5678)
Supplement: Supplementary file 1 [file oncotarget-06-42704-s001.pdf]

## SUPPLEMENTARY FIGURES

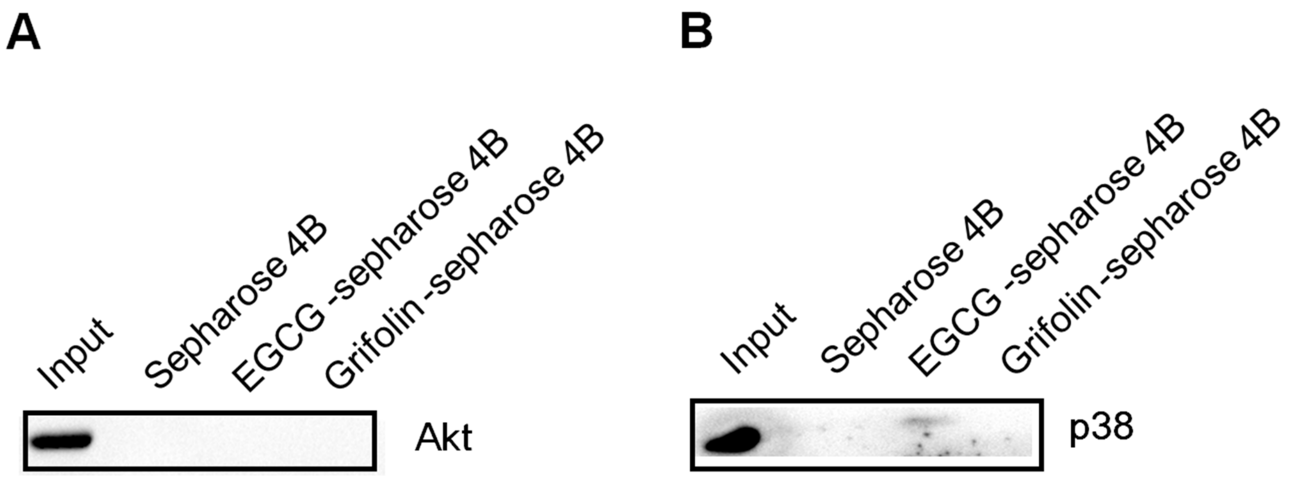


**Supplementary Figure S1: The target specificity of grifolin-binding proteins. Immunoblot analysis of whole cell lysate following purification by a Sepharose 4B, grifolin-Sepharose 4B or EGCG-Sepharose affinity column. A.** The Akt/grifolin complex or **B.** p38/grifolin complex was detected by Western blot using an antibody against Akt and p38, respectively.

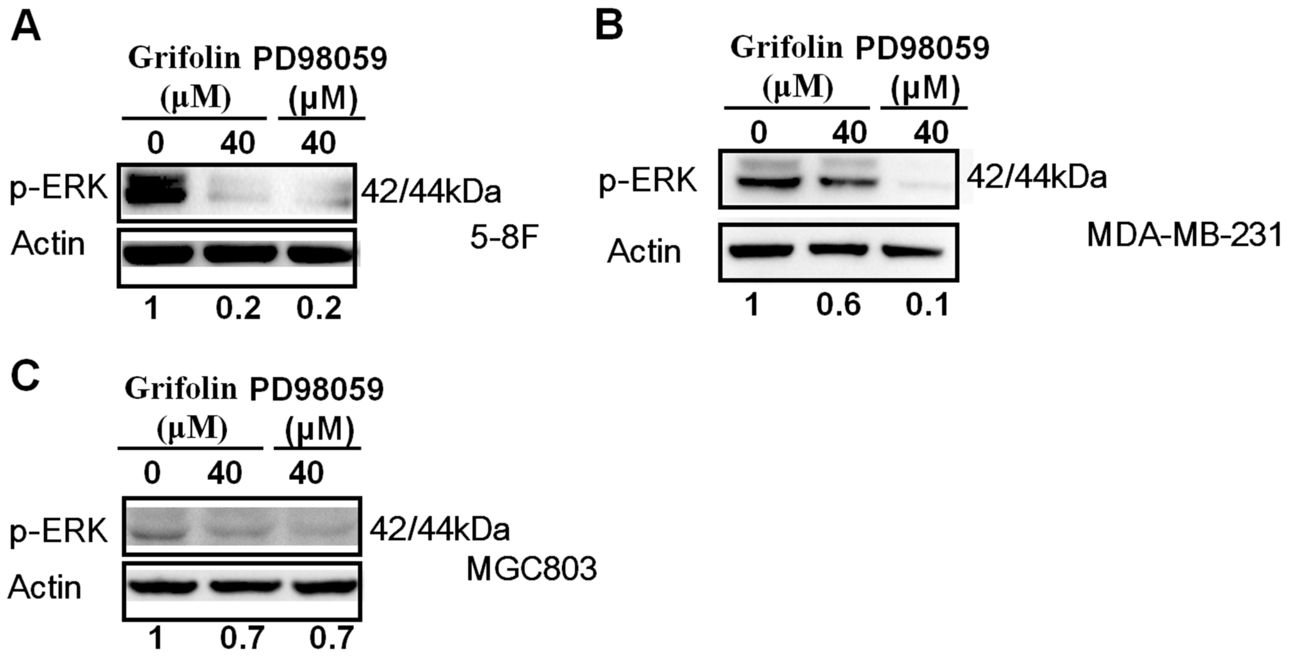


**Supplementary Figure S2: Grifolin decreases phosphor-ERK in high-metastatic cancer cells. A.** 5–8F, **B.** MDA-MB-231 or **C.** MGC803 cells were treated as designated for 12 h. Cell lysates were prepared and examined by Western blot with an antibody against phosphor-ERK.

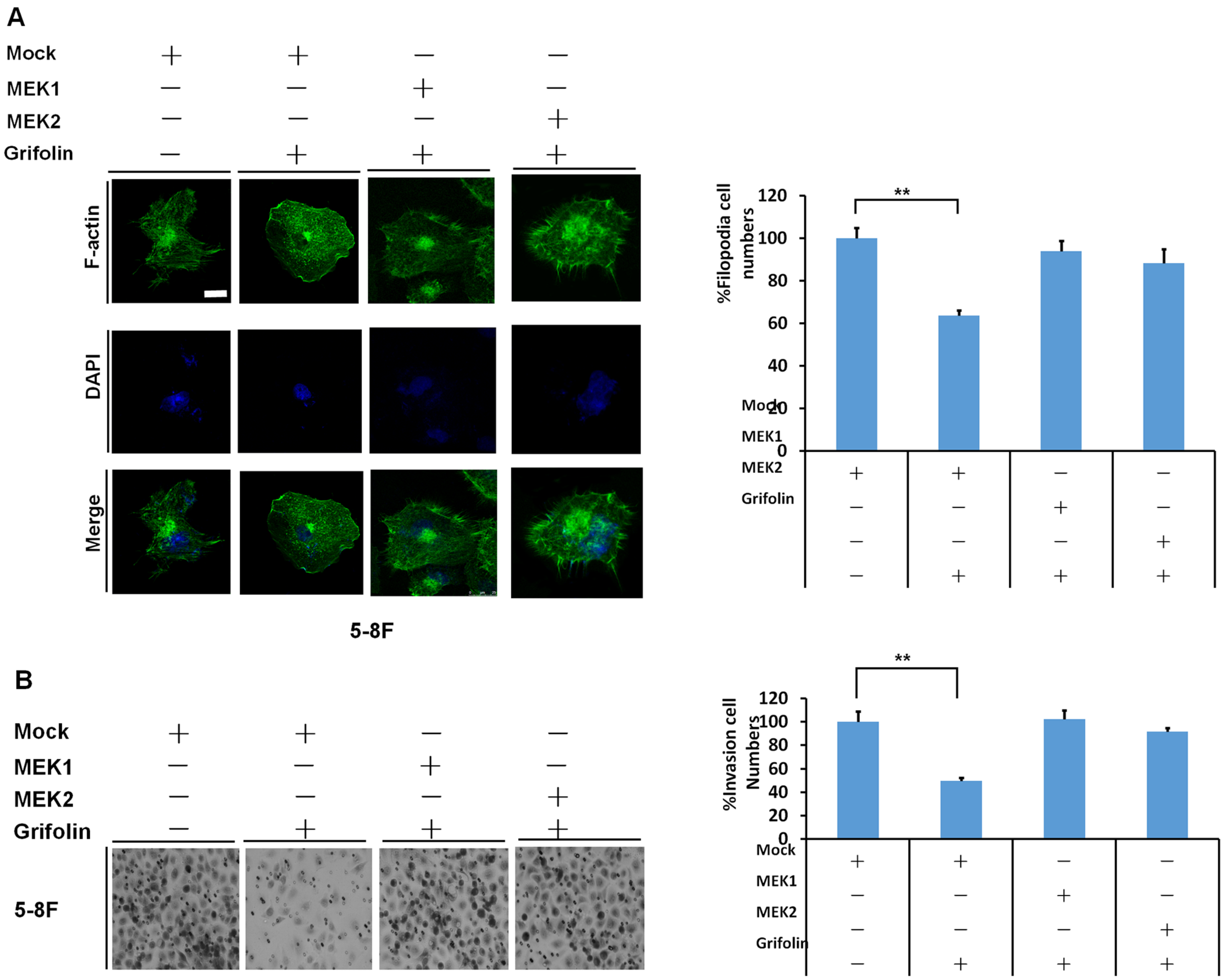


**Supplementary Figure S3: Overexpression of constitutively active MEK1/2 rescue filopodia formation and invasion capacity of 5–8F cells suppressed by grifolin treatment.** 5–8F cells were transfected with constructs as indicated, followed by grifolin or DMSO (control) treatment for additional 24 h, then cells were applied to (A) immunofluorescence analysis or (B) invasion assay. **A.** Cells were stained with fluorescein isothiocyanate/phalloidin and visualized using Leica TCS SP5 confocal microscopy. Images are representative of at least 3 independent experiments. Scale bar, 25 µm. **B.** Counts were obtained (in triplicate fields of view) from the Mock control (average set at 100% invasion) and were used to calculate percent invasion for all other treatments. Data are shown as mean values ± S.D. of independent, triplicate experiments. The asterisks (**) indicate a significant difference ($P < 0.01$) compared to the Mock control.

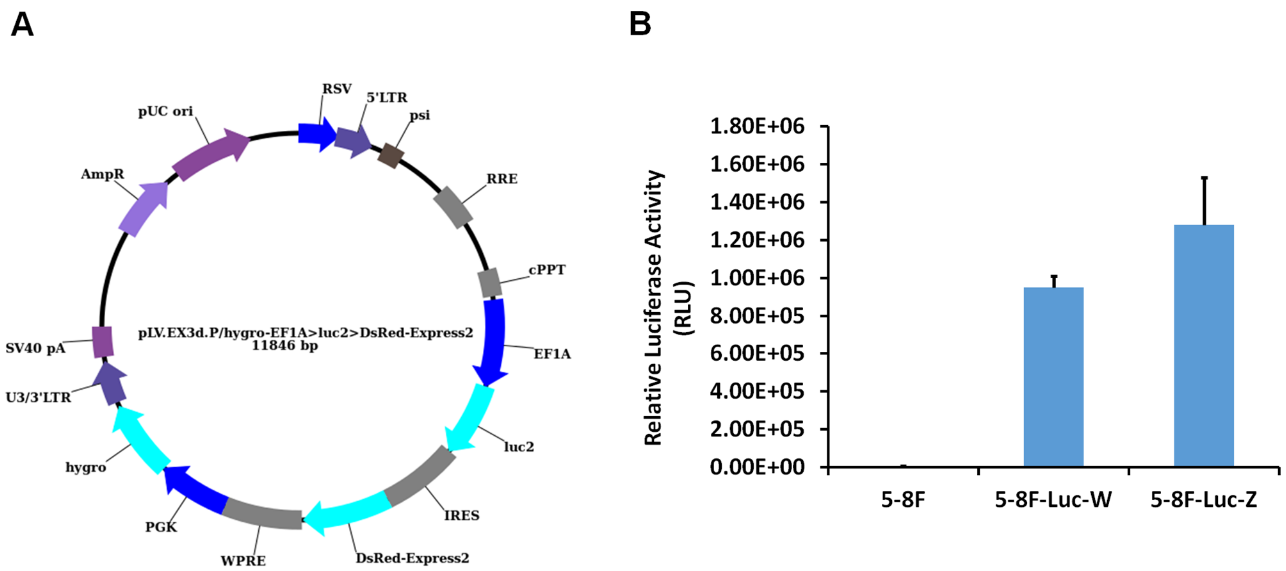


**Supplementary Figure S4: Establishment of nasopharyngeal carcinoma cell 5–8F-Z stable expression of luciferase. A.** The firefly luciferase (luc) gene was inserted into vector pLV.Des3d.P/Hygro by Gateway Recombination Cloning Technology. The construct (pLV.EX3d.P/Hygro-EF1A > luciferase (luc2) > IRES/ DsRed-Express2) was transfected into 5–8F cells to establish two cloned cell line 5–8F-luc-W and 5–8F-luc-Z, which stable expressed luciferase. **B.** The luciferase assay was used to evaluate the luciferase acivity of 5–8F-luc-W and 5–8F-luc-Z, respectively.

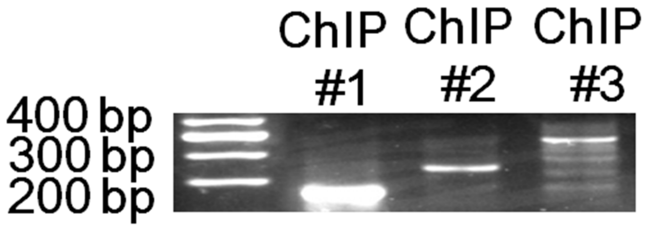


**Supplementary Figure S5: Evaluation efficiency of different ChIP primers for Elk1 binding to the *dnmt1* promoter.** Three pairs of ChIP primers for each potential binding site in the *dnmt1* promoter region were examined for the ability to be enriched by Elk1 immunoprecipitation. ChIP primers were as follows: ChIP 1#, sense 5′-ATCATGG CTCATTGCAGCCTAC-3′; antisense 5′-TCAAGACCAGCCTGAGCAACAT-3′; ChIP 2#, 5′-GCCATG TTG CTC AGGCTGGT -3′; antisense 5′-AACTTAAG GATAGCTATACAGC-3′; ChIP 3#, 5′-GCCATGTTGCTCAGGCTGGT-3′; antisense 5′-AACTATAGTGCTCCTAGAAG-3′. The cross-linked chromatin of 5–8F cell was precipitated with Elk1 antibody. Precipitated DNA was analyzed by PCR using primers that amplified a 158-bp, a 234-bp or a 360-bp sequence corresponding to ChIP 1#, ChIP 2# and ChIP 3#, respectively.

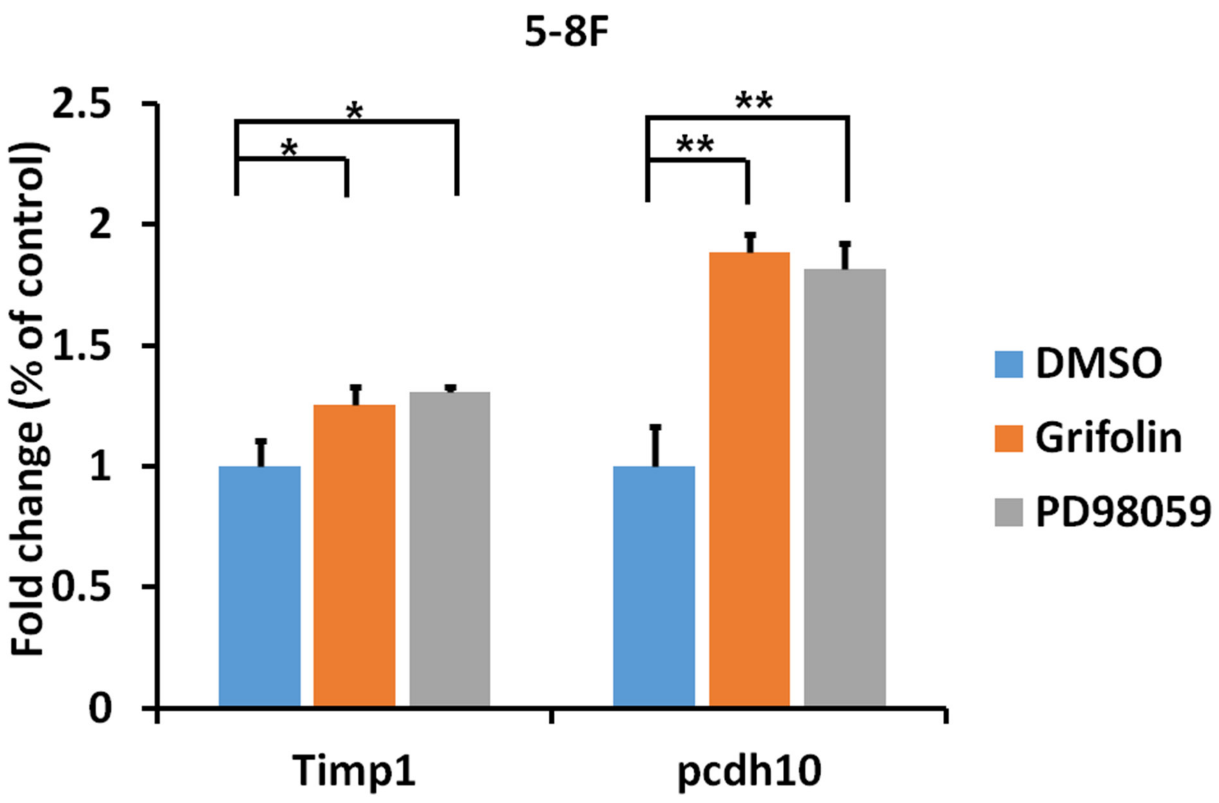


**Supplementary Figure S6: Restored mRNA expression of metastasis suppressor gene Timp1 and pcdh10 induced by grifolin.** 5–8F cells were treated with DMSO, 40 µM grifolin or 40 µM PD98059 as designated for 24 h. Total RNA was isolated from cells and subjected to real-PCR. PCR primers were as follows: Timp1, sense 5′-CTGCGGATACTTCCACAGGTC-3′; antisense 5′-GCAAGAGTCCATCCTGCAGTT-3′; pcdh10, sense 5′-AGTACGGACACTGAGCACAACC-3′; antisense 5′-CGGCGAGGT CTGTCAACTAGATAG-3′. Data are shown as mean values ±S.D. of independent, triplicate experiments. The asterisks (**) indicate a significant difference ($P < 0.01$) compared to the control.
